# Suppression of rice blast and tomato late blight by *Paraboeremia adianticola* producing vulculic acid

**DOI:** 10.1002/ps.8859

**Published:** 2025-05-06

**Authors:** Je‐Hyun Park, Jae Woo Han, Bomin Kim, Sang Un Park, Gyung Ja Choi, Hun Kim

**Affiliations:** ^1^ Center for Eco‐friendly New Materials, Korea Research Institute of Chemical Technology Daejeon South Korea; ^2^ Department of Crop Science Chungnam National University Daejeon South Korea; ^3^ Department of Medicinal Chemistry and Pharmacology University of Science and Technology Daejeon South Korea

**Keywords:** *Paraboeremia adianticola*, vulculic acid, antifungal activity, biological control, mitochondrial respiration

## Abstract

**BACKGROUND:**

In the search for new natural resources with antifungal activity, the culture filtrate of the marine‐derive fungus *Paraboeremia adianticola* SFC20150402‐M24 showed excellent activity for controlling rice blast and tomato late blight diseases. This study aimed to (i) identify antifungal substances from the *P. adianticola* culture filtrate using chromatography and spectroscopy techniques and (ii) investigate the *in vitro* and *in vivo* antifungal activities of the isolated compound, along with its underlying antifungal mechanisms.

**RESULTS:**

Based on chromatographic and spectroscopic analyses, vulculic acid was isolated and identified as a principal antifungal compound of *P. adianticola* SFC20150402‐M24. Among the tested eight fungal pathogens, vulculic acid completely inhibited the growth of *Magnaporthe oryzae* and *Phytophthora infestans* at concentrations of 6.3 and 200 μg mL^−1^, respectively. This inhibition resulted in effective control of rice blast and tomato late blight. Furthermore, given that vulculic acid was more effective in inhibiting spore germination than the mycelial growth of *M. oryzae*, our results showed that vulculic acid has an effect on mitochondrial respiration with the downregulation of *SdhC* and *Cox7A* genes and inhibitory activity for electron transporter chain complexes II, III, and IV.

**CONCLUSION:**

These findings highlight *P. adianticola* SFC20150402‐M24 and its production of vulculic acid as a valuable biological control agent for rice blast and tomato late blight. © 2025 The Author(s). *Pest Management Science* published by John Wiley & Sons Ltd on behalf of Society of Chemical Industry.

## Graphical abstract

This study presents promising results for disease control efficacy using the culture filtrate of *Paraboeremia adianticola* SFC20150402‐M24 and its major active compound, vulculic acid, which affects mitochondrial respiration.

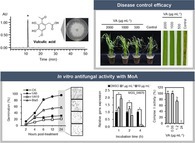

## 1 INTRODUCTION

The development of fungicides from natural products marks a significant advancement in the sustainable management of plant diseases.^1^ Historically, synthetic chemicals have been the mainstay in controlling fungal pathogens which are responsible for substantial crop losses globally.^2^ However, the occurrence of fungicide resistance reduces the efficacy of the chemicals over time, necessitating the development of new fungicides or the use of fungicides in the mixtures.^3^ The environmental and health concerns associated with synthetic fungicides have driven the search for eco‐friendly alternatives.^4^ Natural products derived from plants and microorganisms offer a promising solution^5^; they include a diverse array of bioactive compounds such as essential oils, phytoalexins, and secondary metabolites that exhibit potent antimicrobial properties.^6^, ^7^ The modes of action of these natural fungicides often involve disrupting the cell envelopes or interfering with the essential metabolic functions of fungal pathogens, thereby providing an effective disease control efficacy.^8^, ^9^ Moreover, their biodegradability and lower risk of resistance development make them integral to sustainable agriculture practices.^1^, ^10^ As research continues to evolve, the potential of natural products in developing new fungicidal formulations is crucial for advancing eco‐friendly and effective crop protection strategies.

Marine fungi are a rich source of bioactive compounds with potential applications in various industries, including pharmaceuticals and agrochemicals.^11^ They produce various secondary metabolites, including terpenes, steroids, polyketides, peptides, alkaloids, and polysaccharides, with antimicrobial, anticancer, antiviral, antioxidant, and anti‐inflammatory activities.^12^ In the field of plant protection, numerous studies have demonstrated the efficacy of secondary metabolites from marine‐derived fungi in combating agricultural pathogens. For example, the culture filtrates derived from *Albifimbria verrucaria*, *Paraconiothyrum sporulosum*, and *Aspergillus spp*., which were isolated from a marine environment, exhibited a promising control efficacy for fungal plant diseases, including tomato late blight, wheat leaf rust, and rice blast.^11^, ^13^, ^14^ Moreover, from marine‐derived fungal species, many antifungal compounds, including mycosporulonol, botrallin, and diorcinol, have been identified and characterized for biological activity.^11^, ^14^ These findings are supported by a comprehensive review systematically analyzing the activities of 198 secondary metabolites from marine fungi against plant pathogenic fungi, highlighting the structural diversity and potential of these natural products.^15^

In our antifungal screening program, the culture filtrate of a marine‐derive fungus strain SFC20150402‐M24 showed excellent activity for controlling rice blast disease. The active compound produced by SFC20150402‐M24 was isolated and identified through a series of chromatography and spectroscopy techniques. The *in vitro* and *in vivo* antimicrobial activities of the isolated compound were investigated to determine its effectiveness as a plant disease control agent. To further understand its efficacy in controlling plant diseases, including rice blast, we investigated the antifungal mechanisms of the isolated compound.

## 2 MATERIALS AND METHODS

### 2.1 Microbial strains and culture conditions

*Paraboeremia adianticola* SFC20150402‐M24 isolated from the brown seaweed *Undaria pinnatifida* was provided by the Marine Bio‐Resource Information System (MBRIS; https://www.mbris.kr). Fungal pathogens used in this study were obtained from the Korean Agricultural Culture Collection (KACC; https://genebank.rda.go.kr): *Alternaria brassicicola* KACC 40036, *Botrytis cinerea* KACC 48736, *Colletotrichum coccodes* KACC 48737, *Cladosporium cucumerinum* KACC 40576, *Cylindrocarpon destructans* KACC 41077, *Fusarium oxysporum* f. *sp. lycopersici* KACC 40043, *Magnaporthe oryzae* KACC 46552, and *Phytophthora infestans* KACC 48738. These fungal species were maintained at 25 °C, except for *B. cinerea* and *P. infestans*, which were grown at 20 °C.^16^

To investigate the optimal culture condition, *P. adianticola* SFC20150402‐M24 was grown in various temperatures (20, 25, and 30 °C) and media (Czapek‐dox agar/broth [CDA/CDB; BD Difco, Franklin Lakes, NJ], potato dextrose agar/broth [PDA/PDB; BD Difco], and malt extract agar/broth [MEA/MEB; 3% malt extract and 0.5% peptone]). All culture media were supplemented with artificial seawater (ASW; 2.81% NaCl, 0.07% KCl, 0.16% CaCl_2_∙2H_2_O, 0.48% MgCl_2_∙6H_2_O, 0.713%, 0.11% NaHCO_3_, and 0.35% MgSO_4_∙7H_2_O) as needed.

### 2.2 Isolation, identification, and quantitative analysis of the antifungal compound

To isolate an active antifungal compound from the culture filtrate of *P. adianticola* SFC20150402‐M24, 20 mycelial disks (6 mm diameter) of strain SFC20150402‐M24 grown on PDA were inoculated into 400 mL of PDB in a 2 L‐Erlenmeyer flask and then incubated at 25 °C with agitation at 150 r/min. A 10‐day‐old fungal culture (1.2 L) of SFC20150402‐M24 was filtered with two layers of filter paper and then partitioned with an equal volume of ethyl acetate (EtOAc) and n‐butanol (BuOH), sequentially. EtOAc and BuOH were purchased from Daejung Chemical (Siheung, Korea). Each layer was concentrated to dryness, yielding residues of 1.1 g from the EtOAc layer, 1.9 g from the BuOH layer, and 6.4 g from the water‐soluble layer. The combined EtOAc and BuOH extracts were further purified by the Biotage Isolera One medium‐pressure liquid chromatography system (Uppsala, Sweden) equipped with the Biotage SNAP Ultra C18 50 g cartridge. The column was eluted with a gradient from 20 to 100% aqueous methanol, yielding 300 mg of pure compound **1**. The chemical structure of compound **1** was confirmed by spectroscopic analyses and comparisons with data in the literature.^17^ The electrospray ionization mass spectrometry (ESI‐MS) was recorded on a single‐quadruple mass spectrometer (Acquity QDa; Waters, Milford, MS, USA). The ^1^H and ^13^C nuclear magnetic resonance (NMR) spectra were recorded by a Bruker Advance 500 MHz spectrometer (Bruker BioSpin, Rheinstetten, Germany) in methanol‐*d*_*4*_ (99.8 atom% D; Cambridge Isotope Laboratories, Tewksbury, MA, USA). Chemical shifts were referenced to the solvent peaks (*δ*_H_ 3.31 ppm and *δ*_C_ 49.0 ppm).

For the quantitative analysis of compound **1** in the *P. adianticola* SFC20150402‐M24 culture filtrate, an aliquot (20 μL) of the culture filtrates derived from 7‐day‐old cultures was directly injected into a Waters 515 pump system equipped with the Waters 996 photodiode array detector. The mobile phases were prepared by mixing deionized water and methanol containing 0.1% formic acid. Methanol and formic acid were purchased from Avantor (Radnor, PA, USA). An Agilent Pursuit XRs C18 column (250 × 4.6 mm, 5 μm; Agilent Technologies, Santa Clara, CA, USA) was used, and the flow rate was kept at 1.0 mL/min. The elution was performed as follows: 0 to 5 min, an isocratic elution of 20% methanol; 5 to 25 min, a linear gradient from 20 to 60% methanol; and an isocratic elution of 60% methanol for an additional 10 min. To detect compound **1**, UV absorbance at a wavelength of 254 nm was monitored. The quantity of compound **1** was determined by comparing the peak areas of the sample with those of the standard compound **1**.

### 2.3 Broth microdilution assay for *in vitro* antifungal activity

Minimum inhibitory concentration (MIC) values of the purified compound **1** against phytopathogenic fungi were determined by the broth microdilution assay using the 2‐fold serial dilution method.^18^ Briefly, a spore suspension (1 × 10^5^ spores mL^−1^ of PDB) of each fungal pathogen was added to the wells of a 96‐well microtiter plate. The stock solutions of compound **1** dissolved in dimethyl sulfoxide (DMSO; Daejung Chemical) were added into the microtiter plate at an initial concentration of 200 μg mL^−1^, and then, 2‐fold serial dilutions were performed. The final concentration of DMSO in each treatment did not exceed 1% (*v/v*). The microtiter plates were incubated for 1 to 3 days, and the MIC values were determined by visual inspection of complete growth inhibition. The chemical blasticidin S and a PDB medium containing 1% DMSO were used as positive and negative controls, respectively. The assay was performed two times with three replicates for each compound at all concentrations investigated.

### 2.4 *In planta* assay for disease control efficacy

The plant disease control efficacy of the *P. adianticola* SFC20150402‐M24 culture filtrate and compound **1** was evaluated against rice blast (RCB; caused by *M. oryzae*) and tomato late blight (TLB; caused by *P. infestans*). As host plants, rice (*Oryza sativa* L, cv. Chucheong) and tomato (*Solanum lycopersicum* cv. Seokwang) were grown in a glasshouse at 25 ± 5 °C for 3 to 4 weeks. Culture filtrate was prepared by filtering the SFC20150402‐M24 cultures grown in a PDB medium for 10 days. Compound **1** dissolved in a DMSO solution was adjusted to the final concentrations of 500, 1000, and 2000 μg mL^−1^. The final concentration of DMSO in each treatment was 1% of the volume. Chemical fungicides (50 μg mL^−1^ of blasticidin S for RCB and 100 μg mL^−1^ of chlorothalonil for TLB) and 1% DMSO solution were used as positive and negative controls, respectively. All the chemical fungicides were purchased from Merck (Rahway, NJ, USA).

Plants were inoculated with plant pathogen 24 h after the culture filtrate or compound **1** treatment. For the development of RCB, two‐ or three‐leaf stages of rice plants were inoculated by spraying with a spore suspension (5 × 10^5^ spores mL^−1^) of *M. oryzae*. The inoculated plants were incubated in a humidified chamber (25 °C) for 24 h, and then, the plants were transferred to a growth chamber (25 °C and 80% relative humidity) for 4 days of incubation. For TLB development, tomato plants in the two‐leaf stage were inoculated by spraying with a zoospore suspension (2 × 10^4^ sporangia mL^−1^) of *P. infestans*. The inoculated plants were incubated in a humidified chamber (20 °C) for 2 days, and then, the plants were transferred to a growth chamber (20 °C) for a 24‐h incubation. After incubating each inoculated plant, disease severity based on the leaf lesion area (%) was evaluated. The disease control efficacy was calculated with the following equation: control efficacy (%) = 100 × [1 – B/A], where A is the mean of the lesion area (%) on the leaves of the control plants, and B is the mean of the lesion area (%) on the leaves of the treated plants.^16^ All experiments were conducted twice, with three replicates for each treatment.

### 2.5 Mycelial growth and spore germination inhibition assay

To investigate the effect of compound **1** on the mycelial growth of *M. oryzae*, a mycelial disc (5 mm in diameter) of *M. oryzae* was inoculated onto rice polish agar (RPA; 32 g rice polish, 10 g dextrose, 12 g agar, and 1‐l distilled water) medium containing 50, 100, and 200 μg mL^−1^ of compound **1**, and then, the radial growth of *M. oryzae* was measured at 7 days postinoculation (dpi). For the inhibitory effect of compound **1** on the spore germination of *M. oryzae*, compound **1** was added to *M. oryzae* spore suspensions (5 × 10^5^ spores mL^−1^ of PDB) at concentrations of 5 and 10 μg mL^−1^ and dispensed into sterile hole slide glasses. Hole slide glasses were placed in a humidified box at 25 °C, and the number of germinated spores was counted by microscopic observation in a total of 100 spores until 24 h. RPA and PDB media containing 1% DMSO were used for the negative control. All experiments were conducted twice with three replicates.

### 2.6 Mitochondrial respiratory inhibitory activity assay

The inhibitory action of compound **1** on mitochondrial respiration was investigated by comparing the growth of *Saccharomyces cerevisiae* grown in two different carbon source media: YG (1% yeast extract and 2% glucose) and NFYG (1% yeast extract and 1% glycerol).^19^ To this end, an *S. cerevisiae* A‐139 cell suspension (OD_600_ = 0.3) grown in each medium was added to the wells of a 96‐well microtiter plate, and then, compound **1** dissolved in DMSO was added in a final concentration of 12.5, 25, and 50 μg mL^−1^. The final concentration of DMSO in each treatment did not exceed 1% (*v/v*). One day after the treatment with compound **1**, the optical density (OD_600_) of each well was measured using a microplate reader (Bio‐Rad, Hercules, CA, USA), and the yeast growth inhibition (%) was calculated as follows: [1 − (OD_600_ of treatment/OD_600_ of control)] × 100. As negative and positive controls, distilled water and a quinone outside inhibitor fungicide, kresoxim‐methyl, were used for this assay, respectively.

To further investigate the inhibitory activity against five electron transport chain complexes I—V, we used the MitoTox Complete OXPHOS Activity Assay kit (Abcam, Cambridge, MA, USA) following the manufacturer's instructions. Briefly, compound **1** was suspended in each assay solution at concentrations of 0.06, 1.2, and 24 μg mL^−1^, and the resulting samples were directly added to each oxidative phosphorylation (OXPHOS) complex. Specific inhibitors for each complex were used as a positive control: rotenone (complex I), thenoyltrifluoroacetone (TTFA, complex II), antimycin A (complex III), potassium cyanide (KCN, complex IV), and oligomycin (complex V). Microplate wells coated with a null capture antibody (complexes I, II, IV, and V) or wells lacking mitochondria (complex III) were used as background controls. All chemical inhibitors were purchased from Merck. After adding compound **1** with the assay solution to the 96‐well microplates, the absorbance of each well was immediately measured using an xMark Microplate Spectrophotometer (Bio‐Rad). Absorbance was recorded at 340 nm for complexes I and V, 600 nm for complex II, and 550 nm for complexes III and IV at intervals of 60 s for 2 h (complex I), 60 s for 1 h (complexes II, IV, and V), and 20 s for 5 min (complex III). The activity of each complex was determined by the rate of change in absorbance after background subtraction. All experiments were conducted twice, with three replicates for each treatment.

### 2.7 Gene expression analysis

To analyze the relative gene expression of the succinate dehydrogenase cytochrome B subunit (*SdhC*; MGG_04876) and mitochondrial cytochrome *c* oxidase subunit VIIa (*Cox7A*; MGG_12467) genes of *M. oryzae*, fungal mycelia were harvested 1, 2, and 4 h after compound **1** treatment on 3‐day‐old cultures grown in complete medium (0.6% yeast extract, 0.6% Casamino Acids, and 1% sucrose)^20^ and used for total RNA isolation with the Easy‐Spin Total RNA Extraction Kit (iNtRON Biotechnology, Seongnam, Korea). First‐strand cDNA was synthesized from the total RNA using SuperScript III First‐Strand Synthesis SuperMix (Invitrogen, Carlsbad, CA, USA) and subsequently used for the qPCR assay with MG 2× qPCR MasterMix (SYBR Green) (MGmed, Seoul, Korea). All qPCR assays were performed using the CFX‐96 Real‐Time PCR Detection System (Bio‐Rad). The expression of each gene was normalized to that of the *β*‐tubulin gene (MGG_00604)^21^ and calculated as the fold change based on the 2^−ΔΔCt^ method. The experiments were repeated two times with three replicates each. All PCR primers used in this study were obtained from Macrogen (Daejeon, Korea) and listed in Table S1.

### 2.8 Statistical analysis

All experiments were performed in triplicate with two runs and expressed as the mean ± standard deviation. Statistical analyses were conducted using a one‐way analysis of variance (ANOVA), followed by Tukey's honestly significant difference (HSD) or Student's *t*‐tests. Significant differences were found when *P* < 0.05.

## 3 RESULTS

### 3.1 Characterization of mycelial growth of *P. Adianticola* SFC20150402‐M24

**Figure 1:**
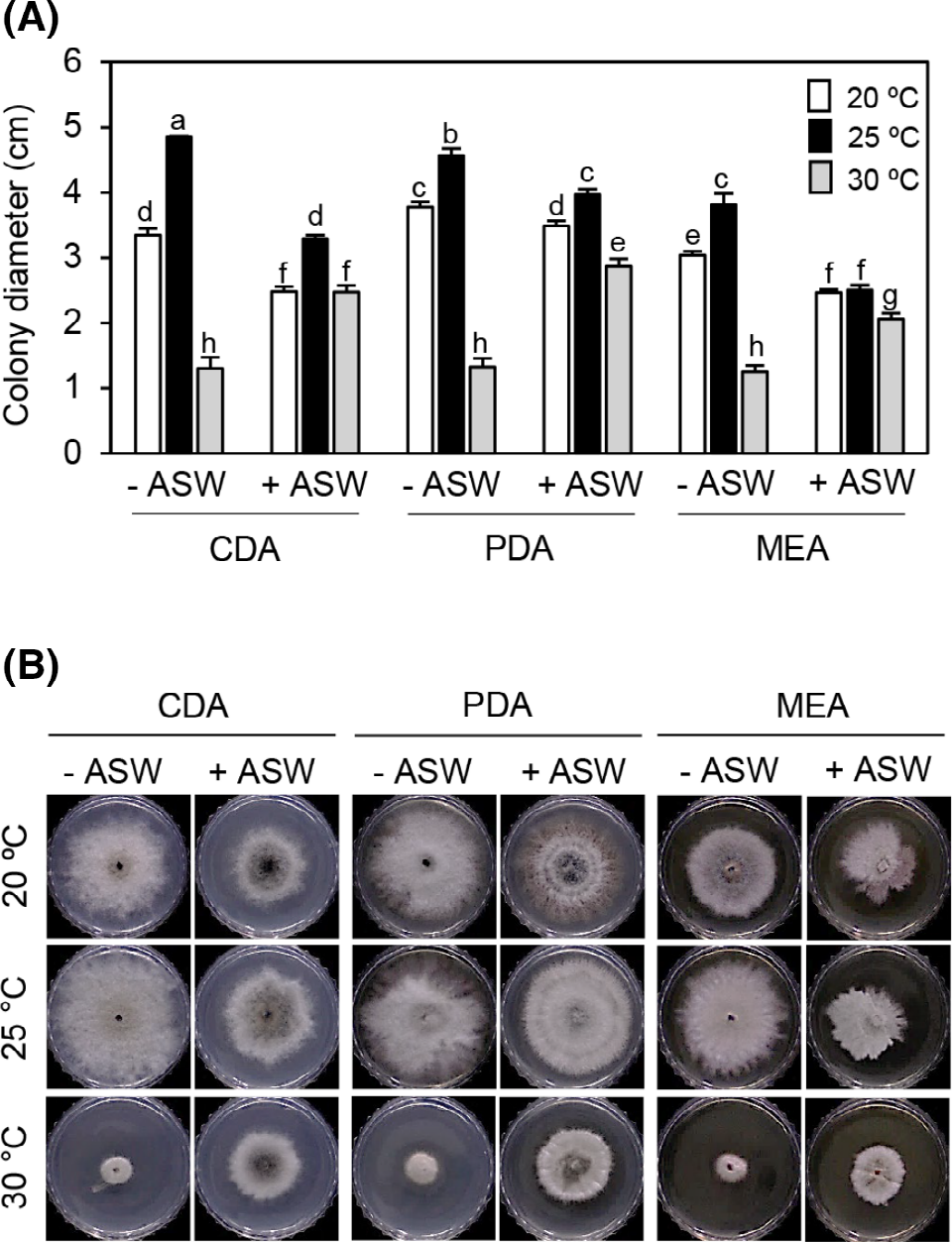
Mycelial growth of *Paraboeremia adianticola* SFC20150402‐M24 on various culture conditions. (A) Colony diameter was measured at 5 days postinoculation. Data are shown as mean ± SD. The different letters above the bar graphs indicate significant differences (Tukey's HSD test; *P* < 0.05). (B) Representative culture plates. CDA, Czapek‐Dox agar; PDA, potato dextrose agar; MEA, malt extract agar; and ASW, artificial seawater.

In the search for useful microbes exhibiting antifungal activity, we discovered that the culture filtrate of strain SFC20150402‐M24 exhibited a potent disease control efficacy for rice blast and tomato late blight (Table S2). Even though the strain SFC20150402‐M24 was identified as *P. adianticola* and provided by the MBRIS, we confirmed in this study by sequence analyses of an internal transcribed spacer region, which showed that the strain was most closely related to the *P. adianticola* CBS 260.92 and CBS187.83 strains with a 100% similarity (Fig. S1). Considering that strain SFC20150402‐M24 was isolated from a marine environment, we investigated how ASW and temperature affect the mycelial growth of the strain. When strain SFC20150402‐M24 was inoculated onto CDA, PDA, and MEA media without ASW, the colony diameter of the strains was maximized at 25 °C on CDA, followed by PDA and MEA (Fig. 1). On all the tested media, strain SFC20150402‐M24 grew well at 20 and 25 °C than at 30 °C. When ASW was added into the medium, the mycelial growth of the strain SFC20150402‐M24 was promoted on all the media at 30 °C (Fig. 1; Table S3). However, at 20 and 25 °C, the strain still grew better on media lacking ASW than on media containing ASW (Fig. 1; Table S3). Therefore, our results suggest that the mycelial growth of strain SFC20150402‐M24 is optimal when cultured on CDA medium without ASW at 25 °C, while ASW supports mycelial growth at relatively higher temperatures.

### 3.2 Chemical identification of the antifungal compound from *P. adianticola* SFC20150402‐M24

**Figure 2:**
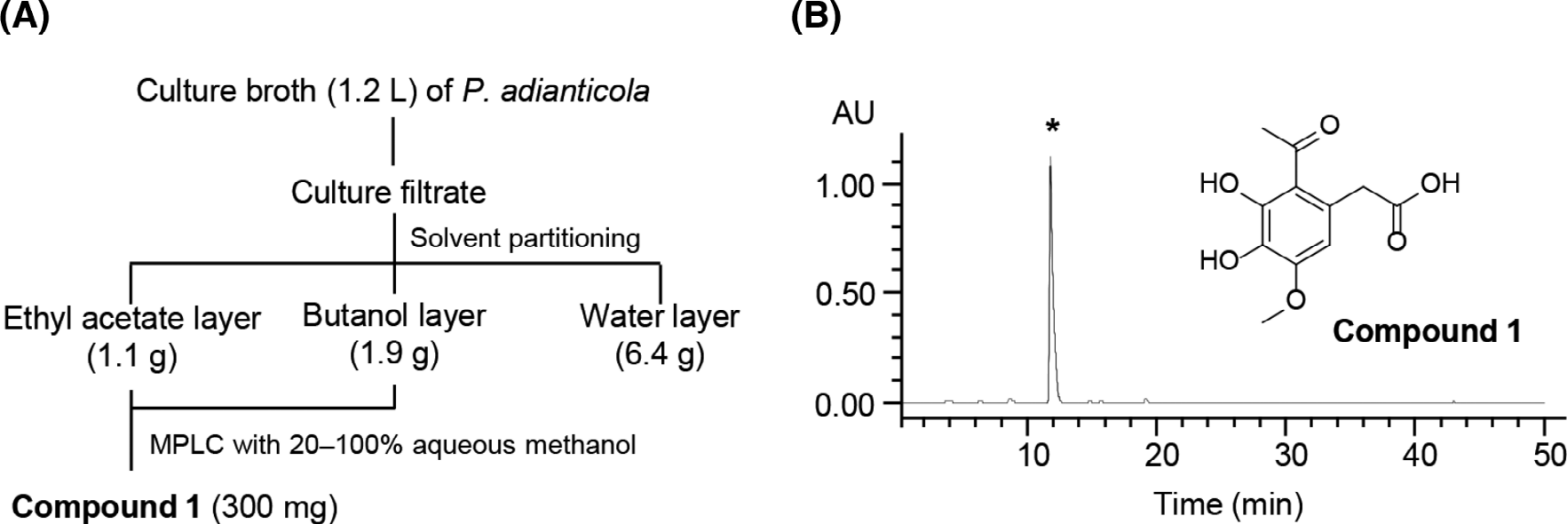
Isolation of an active compound from *Paraboeremia adianticola* SFC20150402‐M24. (A) Isolation scheme and (B) chemical structure of the active compound. The purified active compound peak (*) detected at a retention time of 11.0 min by HPLC analysis.

To isolate an active antifungal compound from the culture filtrate of *P. adianticola* SFC20150402‐M24, a 10‐day‐old fungal culture of SFC20150402‐M24 was sequentially partitioned with EtOAc and BuOH, yielding residues of 1.1 g from the EtOAc layer, 1.9 g from the BuOH layer, and 6.4 g from the water‐soluble layer (Fig. 2(A)). Because the EtOAc and BuOH extracts showed similar patterns in the high‐performance liquid chromatography analysis (Fig. S2(A); Table S4), these extracts were merged and further purified. Consequently, a pure compound **1** (300 mg) was obtained from the culture broth (1.2 L) of *P. adianticola* SFC20150402‐M24 through a series of antifungal activity‐based fractionations (Fig. 2(A)). The molecular mass was determined to be 240 Da by ESI‐MS (m/z 263 [M + Na]^+^) (Fig. S2(B)), and the ^1^H NMR spectrum (Fig. S2(C)) showed a set of four singlet proton signals consisting of an aromatic proton at *δ*_H_ 6.42, an *O*‐methyl proton at *δ*_H_ 3.86, a methylene proton at *δ*_H_ 3.65, and a methyl proton at *δ*_H_ 2.56. The ^13^C NMR spectrum showed two ketone carbonyls (*δ*_C_ 206.4 and 175.8), five quaternary sp^2^ (*δ*_C_ 150.7, 147.4, 134.1, 126.1, and 122.6), a methine sp^2^ (*δ*_C_ 107.6), a methylene sp^3^ (*δ*_C_ 40.1), and two methyl groups (*δ*_C_ 56.5 and 32.3) (Fig. S2(C); Table S5). The spectroscopic data of compound **1** (Fig. S2; Table S5) were consistent with the literature data^17^ for vulculic acid (Fig. 2(B)).

### 3.3 *In vitro* and *in vivo* antimicrobial activity of vulculic acid

To examine the *in vitro* antimicrobial activity spectrum of vulculic acid, we measured the MIC values against each of the eight phytopathogenic fungi and seven bacteria. As shown in Table 1, vulculic acid exhibited antifungal activity against *M. oryzae* and *P. infestans* with MIC values of 6 and 200 μg mL^−1^, respectively; there was no antifungal activity against the remaining six fungal pathogens at a concentration of 200 μg mL^−1^. Additionally, vulculic acid did not exhibit significant levels of antibacterial activity against all the tested plant pathogenic bacteria at a concentration of 200 μg mL^−1^ (Table S6).

**Table 1:** Minimum inhibitory concentrations (MICs) of vulculic acid against plant pathogenic fungi

Fungi	MIC (μg mL^−1^)	
	Vulculic acid	Blasticidin S^a^
*Alternaria brassicicola*	>200	6.3
*Botrytis cinerea*	>200	50
*Cladosporium cucumerinum*	>200	1.6
*Colletotrichum coccodes*	>200	6.3
*Cylindrocarpon destructans*	>200	100
*Fusarium oxysporum*	>200	200
*Magnaporthe oryzae*	6.3	6.3
*Phytophthora infestans*	200	1.6

^a^ Blasticidin S was used as a positive control.

**Figure 3:**
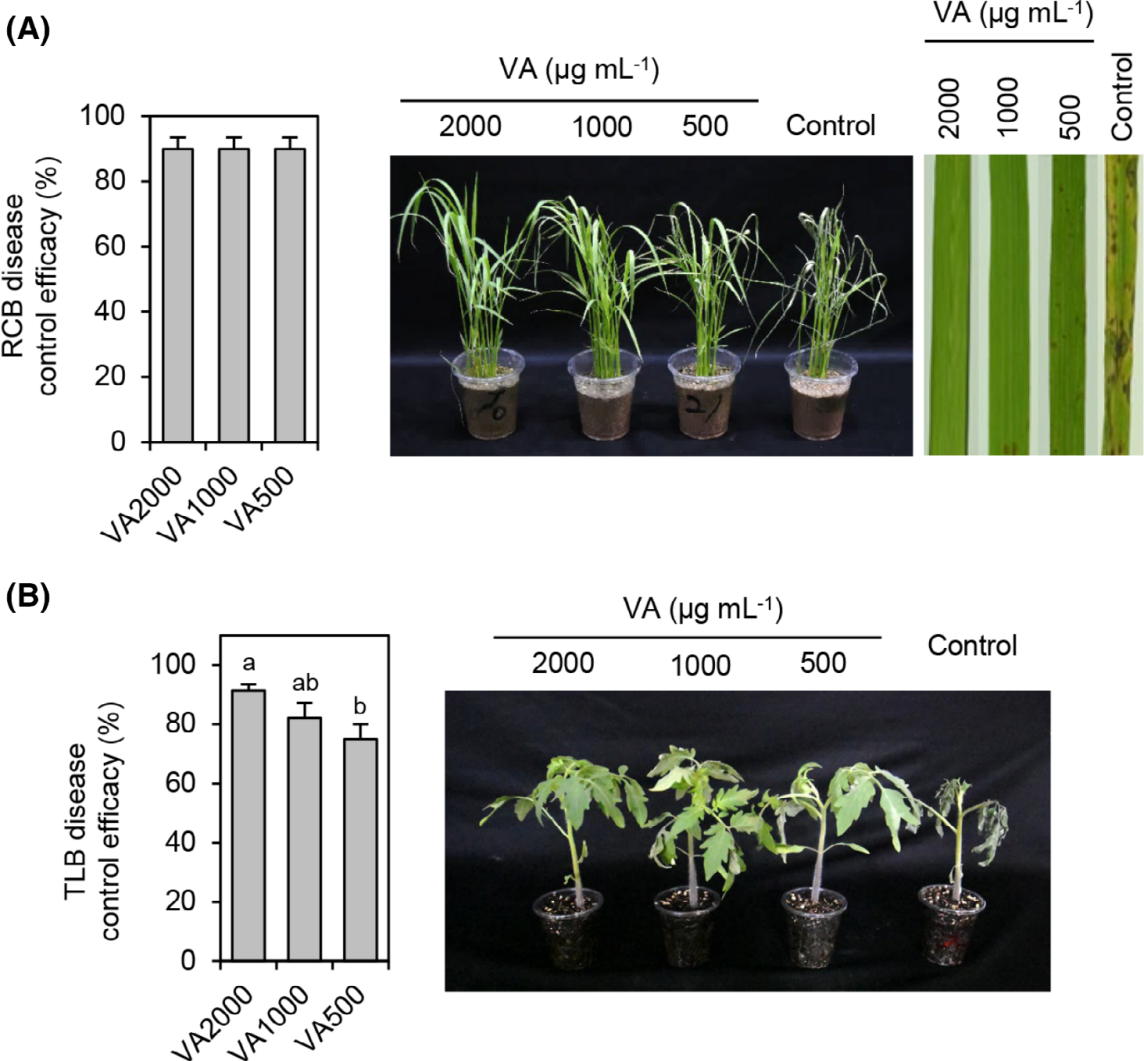
Disease control efficacy of vulculic acid against (A) rice blast (RCB) and (B) tomato late blight (TLB). Control efficacy values were calculated at 3 and 4 days postinoculation for TLB and RCB, respectively. Dimethyl sulfoxide treatment (1%) was used as a control. VA500, VA10000, and VA2000 indicate 500, 1000, and 2000 μg mL^−1^ of vulculic acid (VA), respectively. Data are shown as mean ± SD. The different letters above the bar graphs indicate significant differences (Tukey's HSD test; *P* < 0.05).

Based on the MIC values of vulculic acid, we performed the disease control efficacy assay against rice blast caused by *M. oryzae* and tomato late blight caused by *P. infestans*. When vulculic acid was sprayed on plants before inoculation of the pathogens, vulculic acid exhibited disease control values of over 90% at all the tested concentrations against rice blast disease and also exhibited disease control values of 75, 82, and 91% at concentrations of 500, 1000, and 2000 μg mL^−1^, respectively, against tomato late blight (Fig. 3; Table S7). In addition, no phytotoxicity was observed at the highest treatment concentration of 2000 μg mL^−1^ (Fig. 3).

### 3.4 Vulculic acid content of *P. Adianticola* SFC20150402‐M24 culture filtrates and their plant disease control efficacies

**Figure 4:**
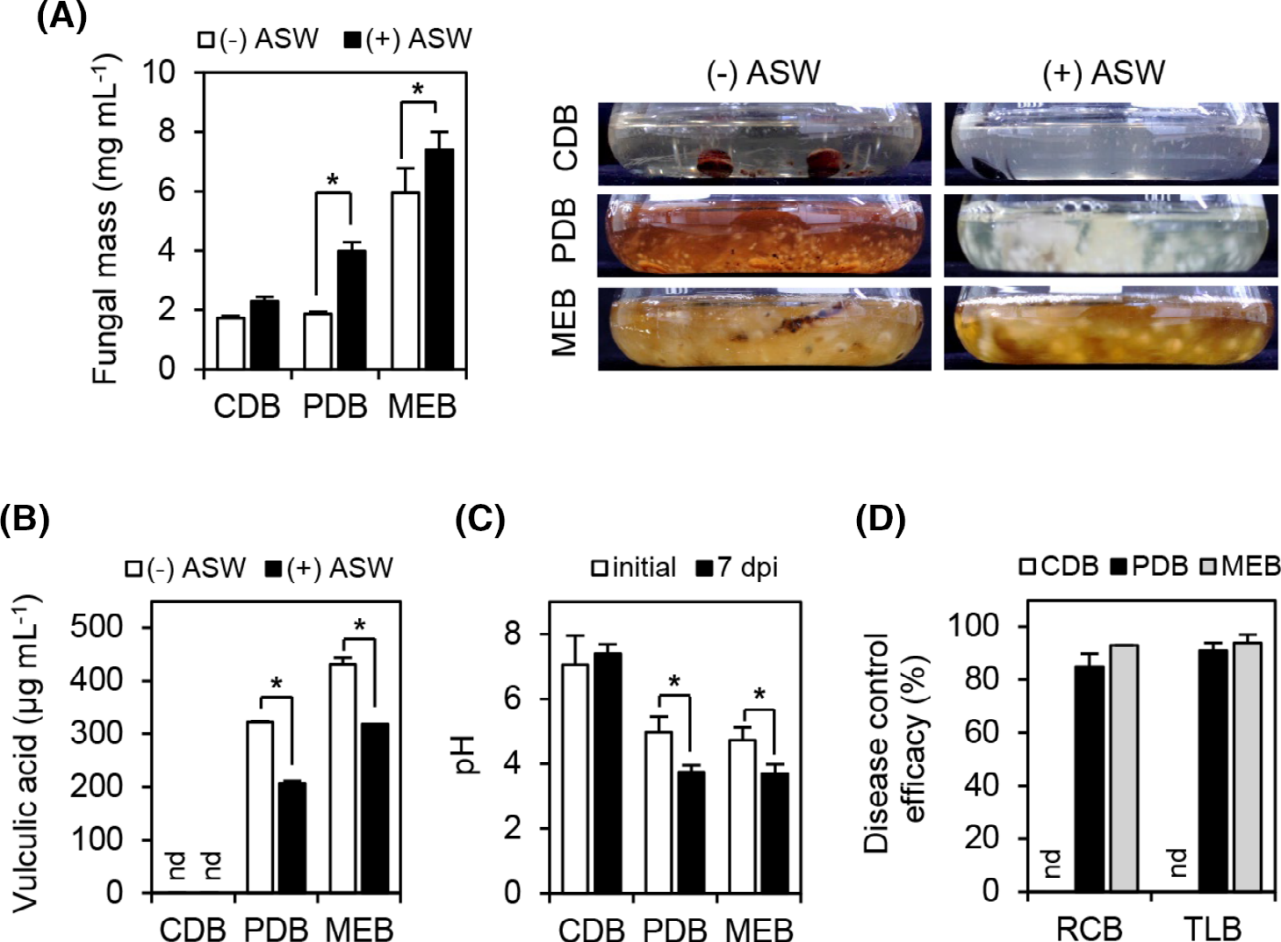
Fungal growth, vulculic acid production, and disease control efficacy of *Paraboeremia adianticola* SFC20150402‐M24 according to culture media. (A) Quantification of fungal dry mass from 7‐day‐old cultures grown at 25 °C; (B) vulculic acid production; (C) pH change; and (D) disease control efficacy of the culture filtrates against rice blast (RCB) and tomato late blight (TLB). CDB, Czapek‐Dox broth; potato dextrose broth; MEB, malt extract broth; and ASW, artificial seawater. Data are shown as mean ± SD. Asterisks indicate a statistically significant difference in mean values at *P* < 0.05 (Student's t‐test). nd, not detected.

To quantify the production yield of vulculic acid from *P. adianticola* SFC20150402‐M24, we inoculated the same amounts of SFC20150402‐M24 mycelial disks into CDB, PDB, and MEB media. Before measuring the vulculic acid amounts, we measured the fungal mass derived from 7‐day‐old CDB, PDB, and MEB cultures. Regardless of the presence or absence of ASW, the dry weight of strain SFC20150402‐M24 was highest in the MEB medium, followed by PDB and CDB (Fig. 4(A)). Unlike growth on a solid medium (Fig. 1), the fungal biomass was higher in the medium containing ASW than in the medium without ASW although the cultures were grown at 25 °C (Fig. 4(A)). When vulculic acid was quantified from the culture filtrates derived from 7‐day‐old cultures of CDB, PDB, and MEB media, we detected 322 and 431 μg mL^−1^ of vulculic acid from the ASW‐free PDB and MEB, respectively; however, there were no detectable levels of vulculic acid from the CDB cultures (Fig. 4(B)). Interestingly, vulculic acid production was decreased in the PDB and MEB media containing ASW, although ASW supports high amounts of fungal biomass in liquid cultures (Fig. 4(B)). Given that strain SFC20150402‐M24 produced vulculic acid in the ASW‐free PDB and MEB media, we measured changes in the pH of the CDB, PDB, and MEB cultures. At 7 dpi, the pH of the PDB and MEB cultures decreased by 1.2 and 1.0 pH units, respectively, whereas the pH of the CDB cultures did not change because there was no vulculic acid production (Fig. 4(C)). Furthermore, when we investigated the disease control efficacy of the culture filtrates derived from 7‐day‐old cultures of the CDB, PDB, and MEB media, the culture filtrates from the PDB and MEB cultures exhibited control values of over 80% against both rice blast and tomato late blight, but the culture filtrate from the CDB culture did not control either disease (Fig. 4(D)). Therefore, our results demonstrate that the cultures of strain SFC20150402‐M24 producing vulculic acid can effectively control rice blast and tomato late blight.

### 3.5 Effect of vulculic acid on the mycelial growth and spore germination of *M. oryzae*

**Figure 5:**
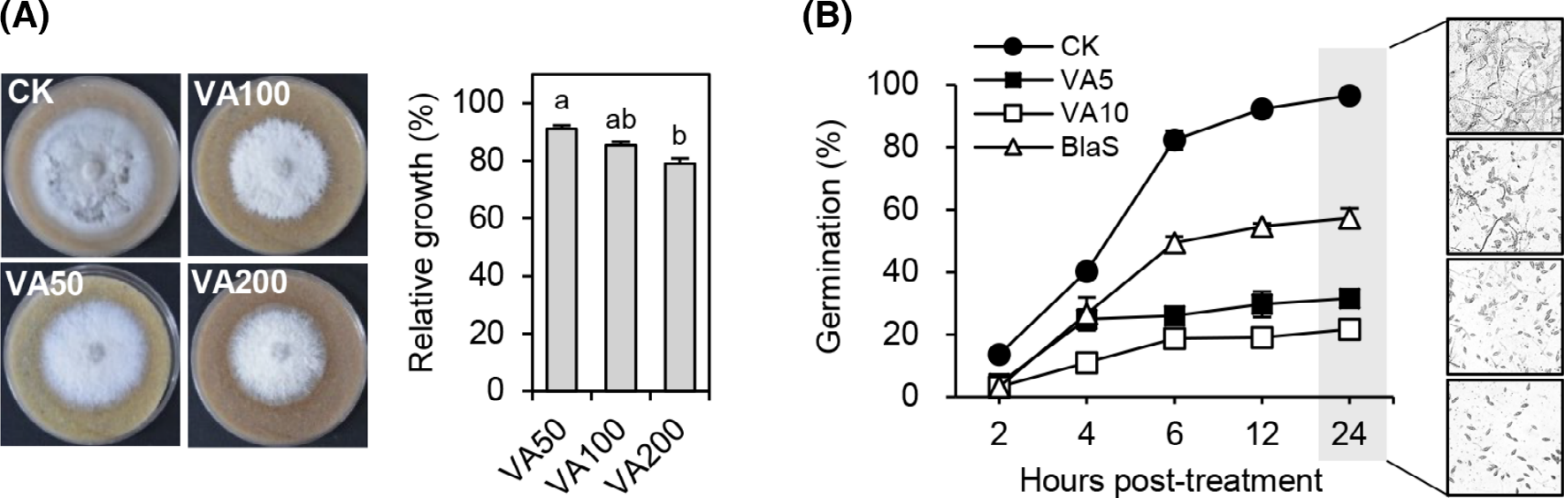
Effects of vulculic acid on (A) mycelial growth and (B) conidial germination of *Magnaporthe oryzae*. Radial growth of *M. oryzae* was measured at 7 days postinoculation. VA5, VA10, VA50, VA100, and VA200 indicate 5, 10, 50, 100, and 200 μg mL^−1^ of vulculic acid, respectively. CK, 1% dimethyl sulfoxide treatment; BlaS, blasticidin S (50 μg mL^−1^). Data are shown as mean ± SD. The different letters above the bar graphs indicate significant differences (Tukey's HSD test; *P* < 0.05).

Based on the lowest MIC value against *M. oryzae* among the tested fungal pathogens, we investigated the effect of vulculic acid on the mycelial growth and spore germination of *M. oryzae*. When *M. oryzae* was grown on an RPA medium supplemented with vulculic acid at concentrations of 50, 100, and 200 μg mL^−1^, the inhibition rate of mycelial growth was 9, 15, and 21% at 7 dpi, respectively (Fig. 5(A)). In contrast, at 6 to 24 h posttreatment of vulculic acid at concentrations of 5 and 10 μg mL^−1^, inhibition of spore germination ranged from 67 to 68% and 77 to 79%, respectively, compared to the non‐treatment control (Fig. 5(B)). Furthermore, vulculic acid exhibited stronger spore germination inhibition than blasticidin S (50 μg mL^−1^), a commercial fungicide used to control rice blast disease (Fig. 5(B)). Taken together, our results show that vulculic acid is much more active in inhibiting spore germination than in inhibiting mycelial growth.

### 3.6 Inhibitory effects of vulculic acid on mitochondrial respiratory complexes

**Figure 6:**
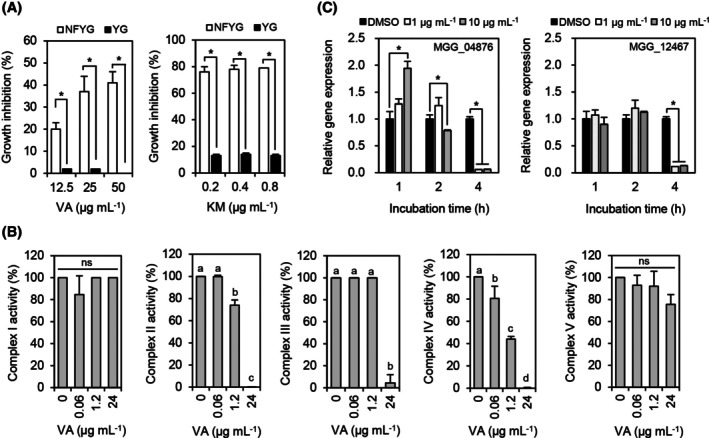
Effect of vulculic acid on mitochondrial respiration. (A) Growth comparison of *Saccharomyces cerevisiae* in NFYG and YG media containing vulculic acid and kresoxim‐methyl. VA, vulculic acid; KM, kresoxim‐methyl. (B) Effect of vulculic acid on mitochondrial respiratory complexes I—V. (C) Effect of vulculic acid on the transcription levels of MGG_04876 (*SdhC*) and MGG_12467 (*Cox7A*) of *Magnaporthe oryzae*. Data are shown as mean ± SD. Asterisks (Student's t‐test) and different letters (Tukey's HSD test in one‐way ANOVA) indicate a statistically significant difference in mean values at *P* < 0.05. ns, not significantly different.

Given that vulculic acid is more effective in inhibiting spore germination than mycelial growth, we hypothesized that vulculic acid has an inhibitory effect on respiration of *S. cerevisiae*. This hypothesis can be supported by previous observations that chemical fungicides targeting mitochondrial respiration exhibit stronger fungicidal activity against spore germination compared to mycelial growth in various filamentous fungi.^9^, ^22^ To determine respiration inhibition activity, many researchers have used a simple assay that compares yeast growth in two different liquid media: one with a fermentable carbon source and the other with a non‐fermentable carbon source as the sole carbon source.^9^, ^19^ When vulculic acid was supplemented in the growth medium at concentrations of 12.5, 25, and 50 μg mL^−1^, it inhibited the growth of *S. cerevisiae* in the range of 20 to 41% compared to the non‐treatment control when they were grown in NFYG, whereas the growth inhibition of *S. cerevisiae* grown in the YG medium ranged from 0 to 2% (Fig. 6(A)). Similarly, treatment with the quinone outside inhibitor fungicide kresoxim‐methyl resulted in greater growth inhibition of *S. cerevisiae* in the NFYG medium compared to the YG medium (Fig. 6(A)). Therefore, we hypothesized that vulculic acid might have an inhibitory activity on fungal respiration. For further details on inhibitory activity of vulculic acid on mitochondrial respiration, we measured the activity of complexes I to V of the respiratory chain in the presence of vulculic acid. Vulculic acid inhibited complexes II, III, and IV with IC_50_ values of 1.7, 7.2, and 1.4 μg mL^−1^, respectively (Fig. 6(B)). The specific inhibitors of the complexes used as positive controls (rotenone, TTFA, antimycin A, KCN, and oligomycin for complexes I, II, III, IV, and V, respectively) also suppressed each complex activity in a concentration‐dependent manner (Fig. S3). Subsequently, when we investigated the effect of vulculic acid on the expression of MGG_04876 (*SdhC*) and MGG_12467 (*Cox7A*) of *M. oryzae* at concentrations of 1 and 10 μg mL^−1^, the expression levels of both *SdhC* and *Cox7A* were significantly decreased by 17 and 8‐fold at 4 h posttreatment, respectively (Fig. 6(C)). Therefore, our results showed that vulculic acid exhibits an inhibitory activity on the respiratory chain complexes.

## 4 DISCUSSION

Chemical control is considered the primary approach to control plant diseases caused by fungi, but the overuse of these fungicides has led to concerns about hazards to humans, animals, and the environment and an increase in fungicide resistance.^3^, ^4^, ^6^ To counteract the escalating risks of chemical fungicides, interest in biological control agents to manage plant diseases has significantly increased. In this study, we found that the culture filtrate of *P. adianticola* SFC20150402‐M24 exhibited a promising disease control efficacy against rice blast and tomato late, with vulculic acid identified as the major antifungal compound in the filtrate. Our observation that vulculic acid was much more effective at inhibiting spore germination of *M. oryzae* than mycelial growth led us to hypothesize that vulculic acid may affect respiratory inhibition. This hypothesis is supported by reports that chemical fungicides targeting mitochondrial respiration show stronger fungicidal activity against spore germination compared to mycelial growth in various filamentous fungi.^9^, ^22^ Our results supported the hypothesis that vulculic acid inhibited the activity of electron transport chain (ETC) complexes II, III, and IV. Mitochondrial respiration is important for spore germination because fungal spore germination requires more adenosine triphosphate (ATP) than mycelial growth.^23^ Spores require a significant amount of energy to break dormancy and initiate growth, relying heavily on mitochondrial respiration for ATP production.^23^ Furthermore, genetic expression during spore germination is highly focused on energy production and cell division, making the spores particularly vulnerable to disruptions in mitochondrial function.^24^

Resistance to single‐site fungicides, including methyl benzimidazoles, demethylation inhibitors, quinone outside inhibitors, and succinate dehydrogenase inhibitors, has been reported in field trials and has spread throughout fungal pathogen populations, reducing the disease control efficacy.^25^, ^26^, ^27^ Despite the crucial role of these fungicides in crop protection from a wide range of fungal diseases,^28^ developing fungicides with novel modes of action could be crucial in crop protection. To our knowledge, no synthetic fungicides targeting mitochondrial respiration *via* cytochrome c oxidase (COX) inhibition have been developed commercially, despite several reports of antifungal compounds that target COX. For example, Zhao *et al*.^29^ synthesized SCU2028, a novel antifungal pyrazole carboxamide containing a diarylamine scaffold, and proposed the mechanism of action of SCU2028, which inhibits complexes II and IV. More recently, Mendes *et al*.^30^ reported that the fungicide cymoxanil inhibits the oxygen consumption of yeast cells and COX activity in the mitochondrial ETC complex IV, suggesting that cymoxanil can be a specific COX inhibitor. Herein, we also found that vulculic acid inhibited the ETC complex IV activity and also down‐regulated *Cox7A* gene expression in *M. oryzae*. These observations and further in‐depth studies on the molecular interactions of vulculic acid with the ETC complex IV could provide insight into the development of COX inhibitors.

In this study, we investigated the production of vulculic acid by *P. adianticola* SFC20150402‐M24 under various culture conditions. Notably, we found that the strain SFC20150402‐M24 produced more vulculic acid in the PDB medium compared to the PDB medium supplemented with ASW. Similarly, the strain produced a significant amount of dark brown pigment in the PDB medium but not in the medium containing ASW. These results can be explained by the fact that the biosynthesis of secondary metabolites is often linked to the production of other bioactive compounds.^31^, ^32^ Notably, adding ASW into liquid media increased the fungal biomass significantly but reduced the vulculic acid production. Salt stress by ASW can have a dual effect on marine fungi, often increasing their biomass while decreasing the production of secondary metabolites.^33^ This phenomenon can be attributed to the osmotic stress imposed by high salt concentrations, which forces fungi to adapt metabolically.^34^ Specifically, fungi may redirect their energy and resources towards growth and maintaining cellular homeostasis, resulting in increased biomass and reduced secondary metabolite production. However, high salinity stress can trigger the activation of silent genes, leading to the production of new secondary metabolites in marine fungi.^33^, ^35^, ^36^, ^37^

Vulculic acid is a polyketide, which was first discovered from the culture filtrate of the fungus *Penicillium sp*. and later from the fungal species *Nimbya alternantherae* and *Chaetosphaeronema achilleae*.^17^, ^38^, ^39^ Regarding biological functions, it has been reported that vulculic acid has antimicrobial, antibiofilm, and cytotoxic activities,^38^ but limited information is available regarding its antimicrobial activity against plant pathogens and plant disease control efficacy. Narmani *et al*.^38^ reported that vulculic acid exclusively exhibited a moderate antimicrobial activity against the yeast *Rhodoturula glutinis* and the Gram‐positive bacterium *Staphylococcus aureus* with MIC values of 67 and 33 μg mL^−1^, respectively, among each of the six tested fungal and bacterial species. In this study, vulculic acid exhibited a weak antifungal activity against *P. infestans* with a MIC value of 200 μg mL^−1^ when we investigated its antimicrobial activity against eight plant pathogenic fungi. However, the rice blast pathogen *M. oryzae* was highly sensitive to vulculic acid with a MIC value of 6 μg mL^−1^; further studies are needed to elucidate why the rice blast fungus *M. oryzae* is particularly sensitive to vulculic acid. Furthermore, the culture filtrate of the strain SFC20150402‐M24 containing vulculic acid suppressed rice blast and tomato late blight. Although it has been reported that vulculic acid inhibits the pollen germination of a pine tree *Pinus thunbergii* and the photosynthetic apparatus of an alligator *weed Alternanthera philoxeroides*,^17^, ^39^ our results showed that vulculic acid was effective in controlling the plant diseases rice blast and tomato late blight without phytotoxicity even at a high concentration of 2000 μg mL^−1^.

Given that vulculic acid exhibited a weak antifungal activity against *P. infestans* with a MIC value of 200 μg mL^−1^, it was unexpected that vulculic acid effectively controlled tomato late blight caused by *P. infestans*. Nevertheless, vulculic acid exhibited disease control values of 75, 82, and 91% at concentrations of 500, 1000, and 2000 μg mL^−1^, respectively, against tomato late blight. We speculated that this effect might be attributed to the mechanism of action of vulculic acid, which inhibits mitochondrial respiration and impairs ATP production. ATP serves as the primary energy source for the flagella that propel the zoospores through their aquatic environment.^40^ Considering that the motility of zoospores is a key factor in the spread of *Phytophthora* diseases, we cannot exclude the possibility that vulculic acid exhibits a promising disease control value against *P. infestans*. Further studies investigating the effect of vulculic acid on zoospore motility will be needed to support this explanation.

In summary, this study demonstrates promising results for disease control efficacy using the culture filtrate of *P. adianticola* SFC20150402‐M24 and its major active compound, vulculic acid. This study highlights its practical potential in crop protection; however, further experiments, including field trials and safety assessments, are needed for practical applications. In addition to its potential as a biological control agent, vulculic acid inhibiting mitochondrial respiration could serve as a lead compound for developing synthetic analogs with enhanced antifungal activity and stability, offering an exciting avenue for developing new fungicides.

## CONFLICT OF INTEREST

The authors declare no conflict of interest.

## Supporting information


**DATA S1:** Supporting Information.

## Data Availability

The data that supports the findings of this study are available in the supplementary material of this article.
