# Supplementary material for: Suppression of rice blast and tomato late blight by Paraboeremia adianticola producing vulculic acid
Source: Pest Manag Sci. 2025 May 6;81(9):5052–61. doi: 10.1002/ps.8859 (PMC12332097; doi:10.1002/ps.8859)
Supplement: Supplementary file 1 — DATA S1: Supporting Information. [file PS-81-5052-s001.pdf]

Supporting Information

Suppression of rice blast and tomato late blight by *Paraboeremia adianticola* producing vulculic acid

Je-Hyun Park,[a,b] Jae Woo Han,[a,c] Bomin Kim,[a,c] Sang Un Park,[b] Gyung Ja Choi[a,c*] and Hun Kim [a,c*]

[a] *Center for Eco-friendly New Materials, Korea Research Institute of Chemical Technology, Daejeon, South Korea*

[b] *Department of Crop Science, Chungnam National University, Daejeon, South Korea*

[c] *Department of Medicinal Chemistry and Pharmacology, University of Science and Technology, Daejeon, South Korea*

Running Title: Biocontrol potential of *Paraboeremia adianticola*

J. Park and J. Han contributed equally to this work

* Correspondence to: Gyung Ja Choi (E-mail: kjchoi@krict.re.kr) and Hun Kim (E-mail: hunkim@krict.re.kr)

Materials and Methods for Supporting Information

Phylogenetic analysis

For the isolation of genomic DNA, strain SFC20150402-M24 was grown in 50 mL of potato dextrose broth (PDB; BD Difco, Flanklin Lakes, NJ, USA) at 25 °C for 4 days on a rotary shaker (150 r/min), and the gDNA was extracted from the harvested mycelia using the cetyltrimethylammonium bromide procedure as previously described.[1] For phylogenetic analysis, the internal transcribed spacer (ITS) region of fungal rDNA was amplified by primer sets ITS1 (5´-TCCGTAGGTGAACCTGCG-3´) and ITS4 (5´-TCCTCCGCTTATTGATATGC-3´). The resulting amplicon was purified using the GeneAll Expin™ PCR purification kit (GeneAll, Seoul, Korea) and then analyzed by a sequencing analysis service (Macrogen, Daejeon, Korea). The resulting sequence was analyzed with the BLASTn program of the NCBI (http://www.ncbi.nlm.nih.gov). The sequences were aligned using ClustalW implemented in MEGA X, and distances were estimated based on the model.[2] A phylogenetic tree was generated using the neighbor-joining method with 1000 bootstrap analyses.

***In vitro* antibacterial activity assay**

The minimum inhibitory concentration (MIC) values of vulculic acid were determined against seven pathogenic bacteria using the broth microdilution method.[3] The plant pathogenic bacteria *A. avenae* subsp. *cattleyyae* SL4351, *Agrobacterium tumefaciens* SL2434, *Burkholderia glumae* SL4269, *Clavibacter michiganensis* SL4135, *Pseudomonas syringae* pv. *actinidiae* CJW7, *Ralstonia solanacearum* SL1944, and *Xanthomonas arboricola* pv. *pruni* SL4370 were provided by the National Academy of Agricultural Sciences (Wanju, Korea) and Dr. Seon-Woo Lee of Dong-A University (Busan, Korea). All bacterial strains were maintained on a tryptic soy agar

(BD Difco). Each bacterial cell was added to the 96-well microtiter plate at a final concentration of 1×10^4 cells mL^{-1}. Vulculic acid (20 mg mL^{-1}) dissolved in dimethyl sulfoxide (DMSO) was serially 2-fold diluted at initial concentrations of 200 µg mL^{-1}. The final concentration of DMSO in each treatment did not exceed 1% (*v/v*). Oxytetracycline and 1% DMSO was used as positive and negative controls. The 96-well plates were incubated for 2–3 days, and the MIC values were determined by visual inspection of complete growth inhibition. Assays were performed two times with three replicates for each treatment.

Disease control efficacy assay

Disease control efficacy of culture filtrates derived from marine-derived fungal isolates were evaluated against rice blast (caused by *Magnaporthe oryzae*), tomato gray mold (caused by *Botrytis cinerea*), tomato late blight (caused by *Phytophthora infestans*), barley powdery mildew (caused by *Blumeria graminis* f. sp. *hordei*), wheat leaf rust (caused by *Puccinia triticina*), and pepper anthracnose (caused by *Colletotrichum coccodes*) as described previously.[4] Briefly, five mycelial disks (8 mm in diameter) of each marine-derived fungus were inoculated into 100 mL of PDB (BD Difco) in a baffled 500 mL Erlenmeyer flask and incubated at 25 °C for 14 days with an agitation of 150 rpm. The resulting culture broth was filtrated through Whatman No. 1 filter paper (Cytiva, Marlborough, MS, USA). For the treatments, the culture filtrate containing 0.025% (*w/v*) Tween 20 solution was sprayed onto the plants. Chemical fungicides (blasticidin-S, fenhexamid, dimethomorph, flusilazole, benomyl, and dithianon) and 0.025% (*w/v*) Tween 20 solution were used as positive and negative controls. As hosts for the pathogens, rice (*Sativa oryza* cv. Chucheong), tomato (*Solanum lycopersicum* cv. Seokwang), wheat (*Triticum aestivum* cv. Geumgang), barley (*Hordeum sativum* cv. Hanyoung), and pepper (*Capsicum annuum* cv.

Hyangchon) were used, which were grown in a greenhouse at 25 ± 5 °C for 2–4 weeks. One day after spraying with the culture filtrate, the treated plants were inoculated with each fungal pathogen and incubated as previously described by Ngo *et al.*[4] The experiment was conducted twice with three replicates for each treatment, and the disease control efficacy was calculated with the following equation: control value (%) = 100 × [1 – B/A], where A is the mean lesion area (%) on the leaves or sheaths of the control plants, and B is the mean lesion area (%) on the leaves or sheaths of the treated plants.

References

1 Oh M, Son H, Choi GJ, Lee C, Kim JC, Kim H et al., Transcription factor ART1 mediates starch hydrolysis and mycotoxin production in *Fusarium graminearum* and *F. verticillioides*. *Mol Plant Pathol* **17**:755–768 (2016).

2 Kumar S, Stecher G, Li M, Knyaz C and Tamura K, MEGA X: molecular evolutionary genetics analysis across computing platforms. *Mol Biol Evol* **35**:1547–1549 (2018).

3 Nguyen MV, Han JW, Kim H and Choi GJ, Phenyl ethers from the marine-derived fungus *Aspergillus tabacinus* and their antimicrobial activity against plant pathogenic fungi and bacteria. *ACS Omega* **7**:33273–33279 (2022).

4 Ngo MT, Han JW, Nguyen MV, Dang QL, Kim H and Choi GJ, Antifungal properties of natural products from *Pterocarya tonkinensis* against phytopathogenic fungi. *Pest Manag Sci* **77**:1864–1872 (2021).

Table S1. Primer list used for RT-qPCR analysis

Gene name (locus no.)	Sequence (5′ to 3′)	
	Forward	Reverse
SdhC (MGG_04876)	CACGTGGTTGTGGTCCAA	CTGCACATTGTGGTAAACGC
Cox7A (MGG_12467)	CTGTACACGTACACCAACGA	GTCAGACCACTCGGGAAAG
β-tubulin (MGG_00604)	ACAACTTCGTCTTCGGTCAG	GTGATCTGGAAACCCTGGAG

Table S2. Disease control efficacy of culture filtrates of marine-derived fungal isolates

Isolates	Control value (%)					
	RCB[a]	TGM	TLB	WLR	BPM	PAN
SFC20150402-M09[b]	0	0	0	0	0	5
SFC20150402-M22	0	0	0	0	0	10
SFC20150402-M04	0	0	0	0	0	0
SFC20150317-M10	13	0	0	0	0	30
SFC20150402-M24	94	0	91	60	0	83
Blasticidin S[c]	100	-	-	-	-	-
Fenhexamid	-[d]	100	-	-	-	-
Dimethomorph	-	-	100	-	-	-
Flusilazole	-	-	-	100	-	-
Benomyl	-	-	-	-	100	-
Dithianon	-	-	-	-	-	95

[a] RCB, rice blast; TGM, tomato gray mold; TLB, tomato late blight; WLR, wheat leaf rust; BPM, barley powdery mildew; and PAN, pepper anthracnose

[b] *Acremonium antarcticum* SFC20150402-M09, *Arthopyrenia salicis* SFC20150402-M22, *Ascochyta manawaorae* SFC20150402-M04, *Botryosporium longibrachiatum* SFC20150317-M10, and *Paraboeremia adianticola* SFC20150402-M24 were provided by the Marine Bio-Resource Information System (https://www.mbris.kr)

[c] Blasticidin S (50 μg mL^{-1}), fenhexamid (100 μg mL^{-1}), dimethomorph (10 μg mL^{-1}), flusilazole (10 μg mL^{-1}), benomyl (100 μg mL^{-1}), and dithianon (50 μg mL^{-1}) were used as positive controls

[d] Not tested

Table S3. Results of three-way ANOVA of SFC20150402-M24 colony diameter affected by medium-type, ASW addition, and incubation temperature

Source of variances	df	*F*	*P*-value	Sig. [a]
Medium type (M)	2	293.553	$< 2e^{-16}$	****
ASW addition (A)	1	44.773	$8.37e^{-08}$	***
Incubation temperature (T)	2	1743.306	$< 2e^{-16}$	****
M × A	2	55.466	$1.01e^{-11}$	***
M × T	4	37.202	$2.53e^{-12}$	***
A × T	2	657.358	$< 2e^{-16}$	****
M × A × T	4	7.204	0.000228	**

[a] Statistical significance level: ns, $P > 0.05$; *, $P < 0.05$; **, $P < 0.001$, ***, $P < 0.0001$; and **** $P < 2e^{-16}$

Table S4. Minimum inhibitory concentrations (MICs) of ethyl acetate (EtOAc) and *n*-butanol (BuOH) layers of *Parboeremia adianticola* SFC20150402-M24 culture filtrate against plant pathogenic fungi

Fungi	MIC (μg mL^{-1})		
	EtOAc layer	BuOH layer	Water layer
Botrytis cinerea	> 1000	> 1000	> 1000
Colletotrichum coccodes	> 1000	> 1000	> 1000
Magnaporthe oryzae	125	125	> 1000
Phytophthora infestans	> 1000	1000	> 1000

Table S5. The ^{13}C NMR spectroscopic data (125 MHz, in CD_3OD) of compound **1** and literature data of vulculic acid

Position	δ_C (ppm)	
	Compound **1** of this study	Vulculic acid[a]
1	175.8	176.5
2	40.1	40.9
3	126.1	126.9
4	107.6	108.5
5	150.7	151.6
6	134.1	134.9
7	147.4	148.2
8	122.6	123.6
9	206.4	207.2
10	32.3	33.1
11	56.5	57.3

[a] Kimura, Y., Nishibe, M., Nakajima, H., and Hamasaki, T. 1991. Vulculic acid, a pollen germination inhibitor produced by the fungus, *Penicillium* sp. Agric. Biol. Chem. 55:1137-1138.

Table S6. Minimum inhibitory concentrations (MICs) of vulculic acid against plant pathogenic bacteria

Bacteria	MIC (μg mL^{-1})	
	Vulculic acid	Oxytetracycline
Acidovorax avenae subsp. *Cattleyae*	> 200	50
Agrobacterium tumefaciens	> 200	3.1
Burkholderia glumae	> 200	1.5
Clavibacter michiganensis	> 200	0.8
Pseudomonas syringae pv. *actinidiae*	> 200	0.8
Ralstonia solanacearum	> 200	6.3
Xanthomonas arboricola pv. *pruni*	> 200	1.5

Table S7. Disease control efficacy of vulculic acid against rice blast (RCB) and tomato late blight (TLB)

Treatment	Concentration (μg mL^{-1})	Control value (%) [a]	
		RCB	TLB [b]
Vulculic acid	500	90 ± 4	91 ± 2 ab
	1000	90 ± 4	82 ± 5 bc
	2000	90 ± 4	75 ± 5 c
Blasticidin S	50	100	–
Dimethomorph	10	– [c]	100 a

[a] ANOVA: df = 3; $F = 5.333$; $P = 0.0698$ for RCB; df = 3; $F = 17.17$; $P = 0.0095$ for TLB

[b] Different letters indicate significant differences between treatments (Tukey's HSD test; $P < 0.05$)

[c] Not tested

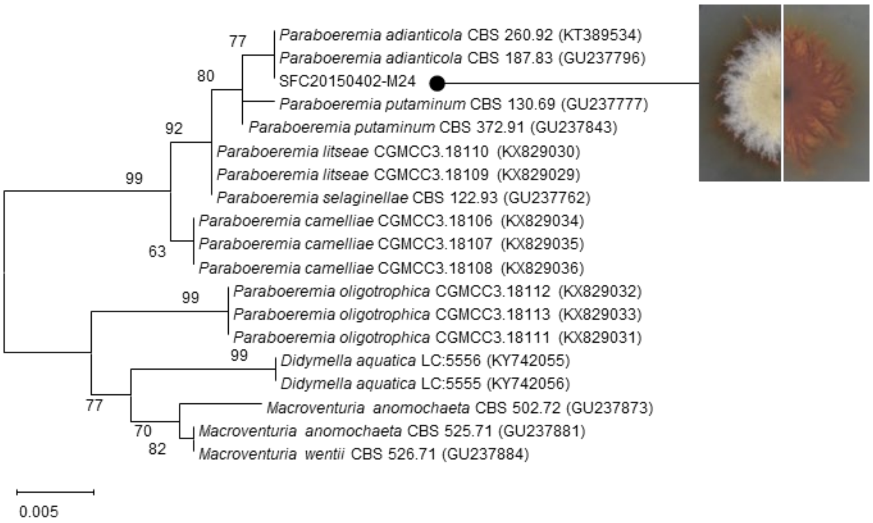

Figure S1. Phylogenetic analysis of the SFC20150402-M24 isolate. Phylogenetic analysis was performed based on the ITS region of SFC20150402-M24 and the other closely-relative species. The neighbor-joining method was used for this analysis, and numbers at the nodes indicate the levels of bootstrap support (%) by 1,000 resampled datasets. NCBI accession numbers of each sequence are in parentheses. Bar, 5 substitution per 1,000 nucleotides. The colony of SFC20150402–M24 had an irregular shape with undulate margins and dark brown pigmentation after 7 days of incubation on potato dextrose agar.

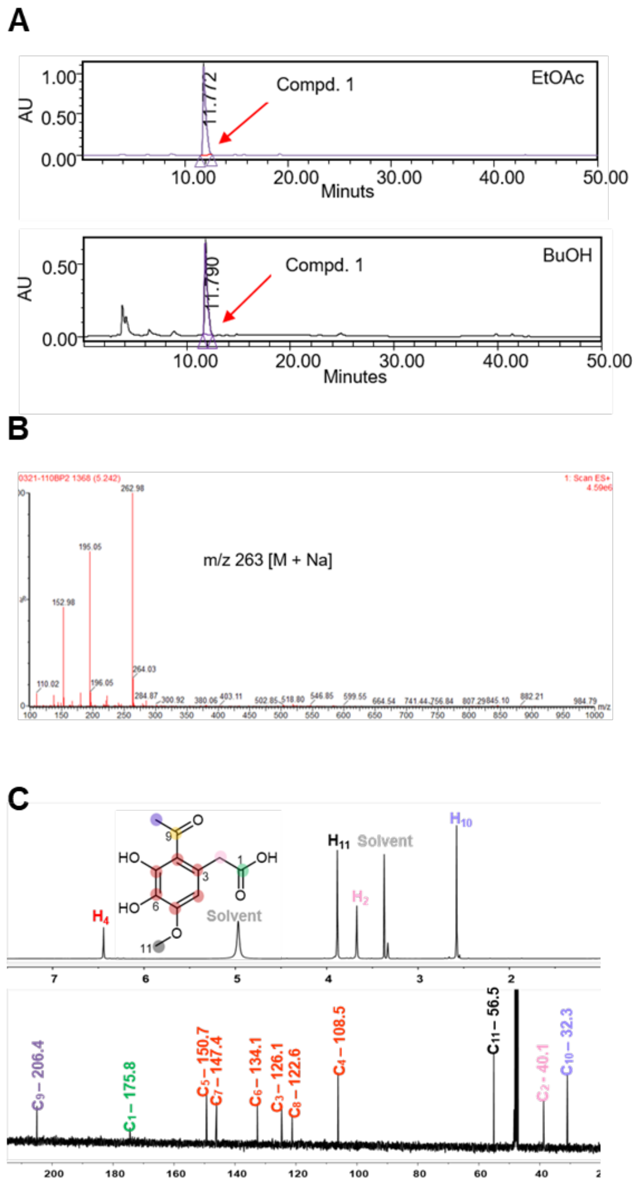

Figure S2. Chromatographic and spectroscopic data for vulculic acid from *Paraboeremia adianticola* SFC20150402-M24**.** Chromatograms (A) for ethyl acetate (EtOAc) and *n*-butanol (BuOH) layers partitioned from culture filtrate of *P. adianticola*, indicates that the major component is compound **1**. ESI-MS data for compound **1** (B) shows a positive ion of m/z 263 [M + Na]. ^{1}H and ^{13}C NMR spectroscopic data (C) indicates that compound **1** is vulculic acid.

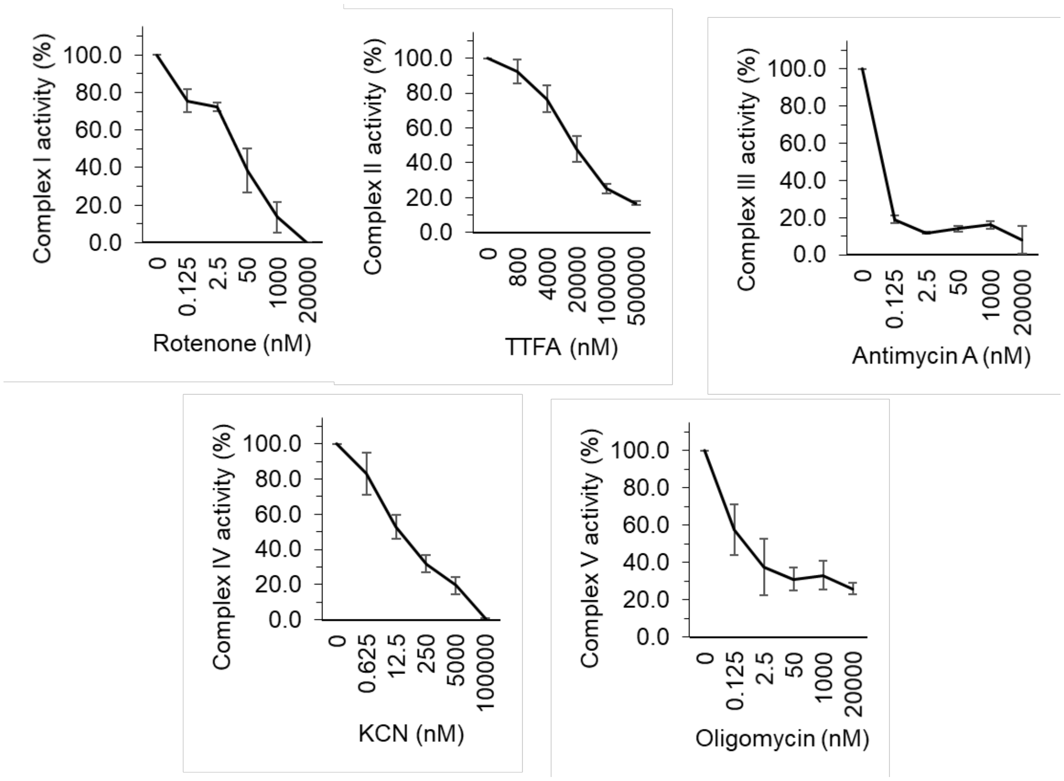

Figure S3. Effect of complex specific inhibitors on the individual mitochondrial respiratory complexes I–V. Rotenone, thenoyltrifluoroacetone (TTFA), antimycin A, potassium cyanide (KCN), and oligomycin were used as positive controls. The experiment was conducted twice with three replicates.
